# Supplementary material for: A Comprehensive Assessment of Ultraviolet-Radiation-Induced Mutations in Flammulina filiformis Using Whole-Genome Resequencing
Source: J Fungi (Basel). 2024 Mar 20;10(3):228. doi: 10.3390/jof10030228 (PMC10971301; doi:10.3390/jof10030228)
Supplement: Supplementary file 1 [file jof-10-00228-s001.zip › Supplementary Material S8/KEGG annotation/out/64381550635650.os/KO/out_map/map05133.html]

KEGG PATHWAY: Pertussis - Reference pathway


|  |  |
| --- | --- |
| **Pertussis - Reference pathway** |  |

[
Pathway menu
| Organism menu
| Pathway entry
| Show description
| User data mapping
]

Pertussis, also known as whooping cough, is an acute respiratory infectious disease caused by a bacteria called Bordetella Pertussis. The characteristic symptoms are paroxysmal cough, inspiratory wheezing and post-tussive vomiting.
Following the inhalation of respiratory secretions from an infected individual, bacteria enter the upper respiratory tract and adhere to epithelial cells. Several adhesion factors have been implicated: the filamentous hemagglutinin (FHA), fimbriae, and pertactin (Prn).
Pertussis toxin (Ptx) and adenylate cyclase toxin (ACT) have been identified so far as major protein toxins of B. pertussis. PTX is a hexameric AB5-type exotoxin. Catalytic A subunit catalyzes the ADP-ribosylation of the Gi subunits of the heterotrimeric G protein, then inhibits multiple downstream pathways. ACT is able to penetrate the cytoplasmic membrane of host cells and becomes activated through the cleavage and the binding of calmodulin (CaM). Activated ACT converts ATP to cyclic AMP and subverts cellular signaling pathways.

|  |  |
| --- | --- |
| Reference pathway | 100% |
